# Finding coevolving amino acid residues using row and column weighting of mutual information and multi-dimensional amino acid representation

**DOI:** 10.1186/1748-7188-2-12

**Published:** 2007-10-03

**Authors:** Rodrigo Gouveia-Oliveira, Anders G Pedersen

**Affiliations:** 1 Center for Biological sequence analysis, The Technical University of Denmark, Building 208, 2800 Lyngby, Denmark

## Abstract

**Background:**

Some amino acid residues functionally interact with each other. This interaction will result in an evolutionary co-variation between these residues – coevolution. Our goal is to find these coevolving residues.

**Results:**

We present six new methods for detecting coevolving residues. Among other things, we suggest measures that are variants of Mutual Information, and measures that use a multidimensional representation of each residue in order to capture the physico-chemical similarities between amino acids. We created a benchmarking system, in silico, able to evaluate these methods through a wide range of realistic conditions. Finally, we use the combination of different methods as a way of improving performance.

**Conclusion:**

Our best method (Row and Column Weighed Mutual Information) has an estimated accuracy increase of 63% over Mutual Information. Furthermore, we show that the combination of different methods is efficient, and that the methods are quite sensitive to the different conditions tested.

## Background

**Figure 1:**
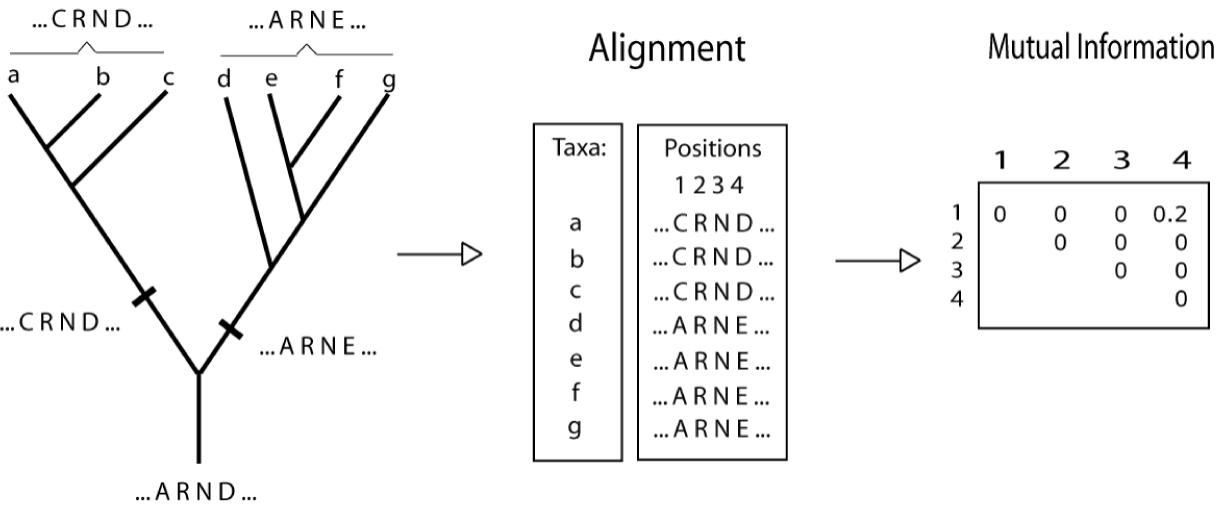
**The Phylogenetic signal mimics coevolution**. A hypothetical phylogeny, the extant sequences and the Mutual Information matrix between the 4 sites of the sequence. Two independent events result in a set of sequences with high mutual information between sites 1 and 4.

Phylogenetic analysis has evolved immensely in the last 30 years, and is now contributing to many exciting discoveries. When performing phylogenetic analysis, it is often assumed that the evolution in one site in a biological sequence is independent of the remaining sites. That makes the task of rebuilding evolution much more tractable and it is in fact a very good assumption in most cases. In some cases, however, sites are not "blind" to a few of the remaining ones. Their evolution is dependent on these other sites. This can occur, for example, when two residues are close in the protein spatial structure, and establish some kind of interaction. In such conditions, the evolution of these sites will depend on each other. A change in one of them will cause the other one to change, and so changes in these sites will occur close in time. This is what we call coevolution. Coevolution has been conceived of as occurring in a variety of settings (see Pollock[1] for a review), including between neighbouring amino acids in a protein's final structure, as compensatory mutations (as in the case of drug-resisting viruses) or between interacting proteins (as in the work of Pazos et al.[2]). But recent findings[3] have shown that coevolving residues seem to be important in the folding of proteins as well, and that they are not necessarily neighbours in the final structure. So the problem "what causes residues to coevolve?" is far from settled. But there is yet another interesting question: "how to find which residues are coevolving?" This question has been addressed in the last ten years by different authors with different methods. The starting point of all these methods is a Multiple Sequence Alignment (MSA). Such an alignment will usually have both conserved and variable sites, with different levels of variability. The alignment will also have some phylogenetic signal. Both these characteristics impose major obstacles on finding coevolving sites. Importantly, it is possible to get perfect co-variation between a pair of non-interacting sites, simply due to the structure of the phylogenetic tree. As sites evolve through a phylogenetic tree, their evolution shares a pattern with the one of coevolution, as can be seen in figure 1.

As an example from comparative biology, where this problem was first identified, lets imagine we wished to compare mammals and birds in many characters, looking for the coevolution in these characters. Their simultaneous absence or presence would be taken as a proof of interaction. We would rightly conclude that having wings has an influence on the presence of light bones, but we would unfortunately also conclude that feathers interacted with laying eggs. In fact, any two characters that segregate according to the phylogeny would be perceived to be coevolving. A pioneering method in extracting the phylogenetic signal in such situations was made by Ridley et al.[4], followed by the one of Maddison[5]. These methods estimate the ancestral state for the characters, and count the co-occurrence of transitions between states. The later method of Harvey and Pagel[6] further incorporated branch length information and was framed as a statistical model. Coming back to sequence analysis, most characters (sites) indeed segregate according to the phylogeny (thus enabling us to reconstruct it), and are therefore hard to separate from truly coevolving sites. One way of going around the problem has been to use highly divergent sequences, in which the phylogenetic signal is weak. However, it is desirable to construct methods that effectively counter this obstacle. The work of Pollock et al.[7] presented one such method, based on likelihood ratio tests. The only obstacle to this methodology is that it is computationally intensive and dependent on the knowledge of the phylogeny. On the other hand, it uses that knowledge to make better predictions. The recent work of Dimmic et al.[8] follows a similar path, but under the Bayesian framework. Most other authors have used simpler approaches, which could eventually be used on large datasets. A majority of these approaches consists of calculating some statistic between all pairs of sites, resulting in a matrix, and these are therefore called "matrix-based methods"[9-17]. Some of these statistics are variations of correlations between some chemico-physical properties of the aminoacids normalized by some quantity, usually their variances[9-11,14,16], others are based on Mutual Information[12,15,18], or in some heuristics not very different from correlation estimation[13]. Exceptions are the methods of Dekker et al.[19] and Suel et al.[20], which are "perturbation-based" methods, in which sub-alignments are build and analyzed. Only a few of these methods, however, tried to tackle the phylogenetic confounding that makes this problem difficult. That was done either by confirming the coevolution predictions in subclades of the tree[17] or by weighting each pair of sites by their phylogenetic dependency[12]. Another main obstacle to accurate coevolution detection has stemmed from biases on site conservation, as shown by Fodor et al.[21]. Recently, however, the conservation bias problem has been addressed [12,15,17,18]. There is the additional problem of not having a good biological control. As we haven't yet answered "what causes residues to coevolve?" we are not able to establish a proper benchmark based on real data. That need for a proper benchmarking platform has caused many artefacts to be published as real findings. We have thus built a complete benchmarking system for this problem, which we use to compare our methods. It has some differences to other benchmarking systems recently made available[13,22]. In this work we simulate alignments of protein sequences including coevolving pairs of sites with differing rates of evolution, differing rates of coevolution, various numbers of taxa and using different methods to simulate coevolution. Simulating these variations is needed to properly evaluate the presented coevolution detecting statistics, as the variations occur in biological datasets and the statistics are sensitive to their effects. It would be more biologically realistic to simulate evolution considering the disturbance to the protein 3D structure, an idea first developed by Parisi and Echave[23] and whose further developments are well described in Rodrigue et al.[24]. We have opted not to do so because the statistics under evaluation do not account for all the extra information available in datasets simulated in this way, but that would definitely be an advantage for more complex methods for detecting coevolution, specially for methods performing n-way comparisons, Such methods would overcome the limitation of detecting n-way coevolution only through its pair wise components, which prevents the detection of networks of sites having many weak pair wise interactions.

## Methods

### Simulated Datasets

Our goal was to compare the performance of the coevolution measures presented in this paper on a number of datasets that covered a wide range of assumptions about how residues evolve and coevolve in real biological data. To do so, we simulated many and varied protein alignments including coevolving residues, applied the above-mentioned methods to find these residues, and then compared their performances. The alignments varied in the number of taxa, in the evolutionary and coevolutionary rates, and in the method of simulating co-evolution. Specifically, each simulated alignment was 300 residues long; 260 residues were independently evolved, while the remaining 40 residues consisted of 20 pairs of coevolving sites. The sequences were simulated along strictly bifurcating trees, of 32, 64, 128 or 256 taxa, with same-size branch lengths. Evolution along the branches followed a BLOSUM62 matrix of transition probabilities[25] – which can be derived from the more common log-odds format – raised to the power r, thus specifying the evolutionary rate. Each alignment had residues evolving at 4 different rates, with values of r of 1 (quickest), 1/5, 1/20 and 1/50 (slowest).

The pairs of coevolving sites were done as described by Pollock et al.[7]. One site was the "driver", evolving as an independent site (also at one of the 4 different rates). The other one was the dependent site. Evolution at the dependent site is, to a level, dependent on the state at the "driver" site. For every state at the "driver" site there is a favoured state at the dependent site. Evolution at the dependent site tends to follow the one at the "driver " site, and this tendency is proportional to a "coevolution factor", c. Coevolution is an easy-to-understand but hard-to-define concept; as we wanted our results to be as general as possible, we modelled coevolution using three different approaches. In all approaches, the evolution at the dependent site was determined by a BLOSUM matrix, transformed by the coevolution factor. The BLOSUM matrix of transition probabilities represents the probability of change from any amino acid (one per row) to any other (one per column). So if we have, for example, a tryptophan as an ancestor, we will use the tryptophan row of the BLOSUM matrix.

*P*(*aa*_*i*_|*w*) = *BLOSUM*_*wi*_

This row will have higher values on the columns corresponding to amino acids similar to tryptophan. On each approach for simulating coevolution, a transformation of the dependent's site BLOSUM matrix by the coevolution factor was applied to the different subsets of columns in the matrix corresponding to a state. That is, the columns belonging to the chosen subset were multiplied by the coevolution factor, thus having their probabilities increased. After the multiplication, the cells were normalized so that the sum of the line still equals one:

P(Depstate|Dep=j&Driver=state)=BLOSUMj,state×coev.f∑iBLOSUMj.i∉state+∑iBLOSUMj,i∈state×coev.f
 MathType@MTEF@5@5@+=feaafiart1ev1aaatCvAUfKttLearuWrP9MDH5MBPbIqV92AaeXatLxBI9gBaebbnrfifHhDYfgasaacH8akY=wiFfYdH8Gipec8Eeeu0xXdbba9frFj0=OqFfea0dXdd9vqai=hGuQ8kuc9pgc9s8qqaq=dirpe0xb9q8qiLsFr0=vr0=vr0dc8meaabaqaciaacaGaaeqabaqabeGadaaakeaacqWGqbaucqGGOaakcqWGebarcqWGLbqzcqWGWbaCdaWgaaWcbaGaem4CamNaemiDaqNaemyyaeMaemiDaqNaemyzaugabeaakmaaeeaabaGaemiraqKaemyzauMaemiCaaNaeyypa0JaemOAaOMaeiOjayIaemiraqKaemOCaiNaemyAaKMaemODayNaemyzauMaemOCaiNaeyypa0Jaem4CamNaemiDaqNaemyyaeMaemiDaqNaemyzauMaeiykaKIaeyypa0dacaGLhWoadaWcaaqaaiabdkeacjabdYeamjabd+eapjabdofatjabdwfavjabd2eannaaBaaaleaacqWGQbGAcqGGSaalcqWGZbWCcqWG0baDcqWGHbqycqWG0baDcqWGLbqzaeqaaOGaey41aqRaem4yamMaem4Ba8MaemyzauMaemODayNaeiOla4IaemOzaygabaWaaabeaeaacqWGcbGqcqWGmbatcqWGpbWtcqWGtbWucqWGvbqvcqWGnbqtdaWgaaWcbaGaemOAaOMaeiOla4IaemyAaKMaeyycI8Saem4CamNaemiDaqNaemyyaeMaemiDaqNaemyzaugabeaakiabgUcaRmaaqababaGaemOqaiKaemitaWKaem4ta8Kaem4uamLaemyvauLaemyta00aaSbaaSqaaiabdQgaQjabcYcaSiabdMgaPjabgIGiolabdohaZjabdsha0jabdggaHjabdsha0jabdwgaLbqabaGccqGHxdaTcqWGJbWycqWGVbWBcqWGLbqzcqWG2bGDcqGGUaGlcqWGMbGzaSqaaiabdMgaPbqab0GaeyyeIuoaaSqaaiabdMgaPbqab0GaeyyeIuoaaaaaaa@A59C@

and thus the dependent site had its evolution conditioned by the "driver" site. The "driver" site "chooses" the amino acids substitutions that will be favoured by increasing the value on the respective columns. The way the subset of columns is defined is what differs between the three coevolution models explained next:

#### 1) Model cluster

The amino acids were grouped into 7 clusters. These clusters were generated by using a non-hierarchical clustering method, k-means, on a transformation of the BLOSUM62 matrix, thus yielding the evolutionarily most meaningful division of amino acids into 7 clusters. They are shown in Table 1.

**Table 1:** Amino acid clusters used in model "Cluster"

**Cluster number:**	**Amino acids**
1	A P S T
2	I L M V
3	F W Y
4	N D
5	G
6	R Q E H K
7	C

These clusters were generated by applying the k-means algorithm to a distance matrix between the amino acids based upon BLOSUM62.

These clusters were the states of the model, defined in equation 2. Each site will evolve according to the row in the BLOSUM matrix corresponding to its own previous/ancestral amino acid. This row, however, will have the columns corresponding to amino acids belonging to group j multiplied by the coevolution factor. That is, if the "driver" site has any amino acid belonging to group j, the dependent site will be pushed into the corresponding state (which for simplicity is also amino acids belonging to group j).

#### 2) Model one-on-one

If Nature wanted amino acids to be clustered into 7 groups, there would only be 7 amino acids, some may say (including ourselves). We therefore also used the one-on-one model of coevolution, where the states are instead the individual amino acids. That is, for each amino acid in the "driver" site there is a favoured one in the dependent site. As we have seen, and for simplicity, we chose it to be the same amino acid, and therefore evolution on the dependent site follows the row of the BLOSUM matrix of the previous/ancestral amino acid, having the column corresponding to the favourite amino acid multiplied by the coevolution factor, c.

#### 3) Model BLOSUM

This model is very similar to the one-on-one. The only difference is the row of the BLOSUM matrix used for determining the probabilities of change. In the previous model, the row was the one of the ancestral amino acid. That is, if the dependent site had an alanine as a residue, and the "driver" site had changed to a proline, the evolution at the end of the branch would follow the BLOSUM row corresponding to the alanine, with an increase probability of changing to a proline. Therefore, it would have high chances of changing into a proline and of staying as an alanine, and mild chances of changing into amino acids similar to alanine. In the BLOSUM model, the BLOSUM row taken is instead the one corresponding to the "driver" site's new amino acid, being given thus more importance to the new physico-chemical demands of that site. In this example, it would be the proline row, having thus increased chances of changing into proline and amino acids similar to proline.

*P*(*Dep*_*state*_|*Dep *= *j *&*Driver *= *i*_{*i*∈*state*}_) = *BLOSUM*_*i, state *_× *coev.f*

This effect, however, should be quite small, as it proved to be. For each model, we tried three different values in the coevolution factor c, yielding a strong, medium or weakly coevolving version. We made 10 replicates of each alignment through all combination of coevolving model, c, and number of taxa. Each alignment had 20 coevolving pairs.

### Analysis

After the alignments had been simulated, we applied the different methods for detecting coevolution to analyze them. All of these methods calculate a statistic for each possible pair of sites in the sequence. Each site is in fact an ordered column of residues. The calculation of a statistic for each pair yields an n by n-1 upper triangular matrix, n being the length of the protein sequence. The cells in the matrix are then ranked and a rank list is presented as the output. The statistics used for each pair wise comparison between sites were:

#### 1) Mutual Information

Mutual Information (MI) is a widely used statistic in several fields, including the present one[26]. It appeared first in Shannon's book/article[27] and has later gained ground as Information Theory developed. It measures the amount of information that one random variable contains about another random variable.

I(A;B)=∑i∑jP(ai,bj)log⁡[P(ai,bj)P(ai)P(bj)]
 MathType@MTEF@5@5@+=feaafiart1ev1aaatCvAUfKttLearuWrP9MDH5MBPbIqV92AaeXatLxBI9gBaebbnrfifHhDYfgasaacH8akY=wiFfYdH8Gipec8Eeeu0xXdbba9frFj0=OqFfea0dXdd9vqai=hGuQ8kuc9pgc9s8qqaq=dirpe0xb9q8qiLsFr0=vr0=vr0dc8meaabaqaciaacaGaaeqabaqabeGadaaakeaacqWGjbqscqGGOaakcqWGbbqqcqGG7aWocqWGcbGqcqGGPaqkcqGH9aqpdaaeqaqaamaaqababaGaemiuaaLaeiikaGIaemyyae2aaSbaaSqaaiabdMgaPbqabaGccqGGSaalcqWGIbGydaWgaaWcbaGaemOAaOgabeaakiabcMcaPiGbcYgaSjabc+gaVjabcEgaNnaadmaabaWaaSaaaeaacqWGqbaucqGGOaakcqWGHbqydaWgaaWcbaGaemyAaKgabeaakiabcYcaSiabdkgaInaaBaaaleaacqWGQbGAaeqaaOGaeiykaKcabaGaemiuaaLaeiikaGIaemyyae2aaSbaaSqaaiabdMgaPbqabaGccqGGPaqkcqWGqbaucqGGOaakcqWGIbGydaWgaaWcbaGaemOAaOgabeaakiabcMcaPaaaaiaawUfacaGLDbaaaSqaaiabdQgaQbqab0GaeyyeIuoaaSqaaiabdMgaPbqab0GaeyyeIuoaaaa@5E93@

where A and B are the two sites being compared, and *i *and *j *run through all the occurring amino acids in each site. The base for the logarithm is 20, the number of letters in the protein alphabet. This causes the MI statistic to scale to a maximum of 1. To estimate MI, here and in the following sections, we have estimated all the probabilities involved by their correspondent observed frequencies.

#### 2) Mutual Information of adaptable logarithmic base

MI between two columns in an alignment reaches the maximum value of 1 if and only if three conditions are met. First, that there is perfect co-variation between the residues present in two sites. Second, that all the 20 different amino acids occur in the two sites (this is due to the logarithmization of base 20). And finally, that the 20 different amino acids are present in equal frequencies. The MI score between two pairs of sites is dependent on these three conditions. We believe that only the first is a desirable property for a measure of coevolution. The other two properties, even though it can be argued to carry some desired coevolution effects, are also prone to raise the MI score of false positives. If one uses MI to estimate coevolution on a simulated dataset without any coevolving sites, one will obtain a clique of the quickest evolving sites coevolving with each other (data not shown). These characteristics of MI seem to not have been noticed by most previous publications, such as[28], until it was specifically addressed by Martin et al.[15]. To counter the second condition, we propose the Mutual Information of Adaptable Logarithmic Base (MI Adp):

I(A;B)=∑i∑jP(ai,bj)log⁡[P(ai,bj)P(ai)P(bj)]/log⁡(#i⋅#j)
 MathType@MTEF@5@5@+=feaafiart1ev1aaatCvAUfKttLearuWrP9MDH5MBPbIqV92AaeXatLxBI9gBaebbnrfifHhDYfgasaacH8akY=wiFfYdH8Gipec8Eeeu0xXdbba9frFj0=OqFfea0dXdd9vqai=hGuQ8kuc9pgc9s8qqaq=dirpe0xb9q8qiLsFr0=vr0=vr0dc8meaabaqaciaacaGaaeqabaqabeGadaaakeaacqWGjbqscqGGOaakcqWGbbqqcqGG7aWocqWGcbGqcqGGPaqkcqGH9aqpdaWcgaqaamaaqababaWaaabeaeaacqWGqbaucqGGOaakcqWGHbqydaWgaaWcbaGaemyAaKgabeaakiabcYcaSiabdkgaInaaBaaaleaacqWGQbGAaeqaaOGaeiykaKIagiiBaWMaei4Ba8Maei4zaC2aamWaaeaadaWcaaqaaiabdcfaqjabcIcaOiabdggaHnaaBaaaleaacqWGPbqAaeqaaOGaeiilaWIaemOyai2aaSbaaSqaaiabdQgaQbqabaGccqGGPaqkaeaacqWGqbaucqGGOaakcqWGHbqydaWgaaWcbaGaemyAaKgabeaakiabcMcaPiabdcfaqjabcIcaOiabdkgaInaaBaaaleaacqWGQbGAaeqaaOGaeiykaKcaaaGaay5waiaaw2faaaWcbaGaemOAaOgabeqdcqGHris5aaWcbaGaemyAaKgabeqdcqGHris5aaGcbaGagiiBaWMaei4Ba8Maei4zaC2aaeWaaeaadaGcaaqaaiabcocaJiabdMgaPjabgwSixlabcocaJiabdQgaQbWcbeaaaOGaayjkaiaawMcaaaaaaaa@6B1D@

where #i represents the number of different amino acids occurring in site A, and #j in site B.

By using a logarithmic base, which is the geometric average of the number of different amino acids present in each site, we make it possible for every score to range from 0 to 1, independently of the number of different amino acids present in the two original sites.

#### 3) Simple correlation

The MI Adp measure is independent of the number of different amino acids present in each pair of sites, but will still favour sites in which the different amino acids occur at even frequencies. That is a consequence of the logarithmization, as the function

−∑in(xlog⁡(x))
 MathType@MTEF@5@5@+=feaafiart1ev1aaatCvAUfKttLearuWrP9MDH5MBPbIqV92AaeXatLxBI9gBaebbnrfifHhDYfgasaacH8akY=wiFfYdH8Gipec8Eeeu0xXdbba9frFj0=OqFfea0dXdd9vqai=hGuQ8kuc9pgc9s8qqaq=dirpe0xb9q8qiLsFr0=vr0=vr0dc8meaabaqaciaacaGaaeqabaqabeGadaaakeaacqGHsisldaaeWaqaaiabcIcaOiabdIha4jGbcYgaSjabc+gaVjabcEgaNjabcIcaOiabdIha4jabcMcaPiabcMcaPaWcbaGaemyAaKgabaGaemOBa4ganiabggHiLdaaaa@3CCF@

with 0<x<1 has its maximum at x = 1/n. To use a measure that is solely dependent on the correlation between sites, we simply used:

I(A;B)=∑i∑j[P(ai,bj)2P(ai)P(bj)]
 MathType@MTEF@5@5@+=feaafiart1ev1aaatCvAUfKttLearuWrP9MDH5MBPbIqV92AaeXatLxBI9gBaebbnrfifHhDYfgasaacH8akY=wiFfYdH8Gipec8Eeeu0xXdbba9frFj0=OqFfea0dXdd9vqai=hGuQ8kuc9pgc9s8qqaq=dirpe0xb9q8qiLsFr0=vr0=vr0dc8meaabaqaciaacaGaaeqabaqabeGadaaakeaacqWGjbqscqGGOaakcqWGbbqqcqGG7aWocqWGcbGqcqGGPaqkcqGH9aqpdaaeqaqaamaaqababaWaamWaaeaadaWcaaqaaiabdcfaqjabcIcaOiabdggaHnaaBaaaleaacqWGPbqAaeqaaOGaeiilaWIaemOyai2aaSbaaSqaaiabdQgaQbqabaGccqGGPaqkdaahaaWcbeqaaiabikdaYaaaaOqaaiabdcfaqjabcIcaOiabdggaHnaaBaaaleaacqWGPbqAaeqaaOGaeiykaKIaemiuaaLaeiikaGIaemOyai2aaSbaaSqaaiabdQgaQbqabaGccqGGPaqkaaaacaGLBbGaayzxaaaaleaacqWGQbGAaeqaniabggHiLdaaleaacqWGPbqAaeqaniabggHiLdaaaa@5227@

Which can also be seen as MI without the logarithmization. This measure has the desirable property of being only dependent on the correlation between the sites. A possible problem is that by neglecting the diversity of amino acid composition, it will score very conserved pairs highly, including the totally conserved sites. Thus, for totally conserved sites we, a posteriori, assigned the value 0 to the estimator, as these conserved sites are obviously not coevolving.

#### 4) Row-column weighting

Let us think of every site in the alignment as a column-vector. If the sequences had no evolutionary signal and there were no constraints, the vector would approach n random realizations from a multinomial distribution with probabilities reflecting the amino acid composition. For a sufficiently high n (number of sequences), the chance of a vector of length n having the same pattern of conservation as another site would then be very small, and no false positives would result.

However, when there is evolutionary signal, sequences that are more closely related will tend to have the same or similar amino acids when compared with more distantly related sequences. This means that some patterns of amino acid conservation are much more common in the alignment columns than others. This is, in fact, the reasoning behind the construction of phylogenetic trees: sites are grouped into patterns, and the resulting tree is the one which is compatible with the majority of observed patterns.

**Figure 2:**
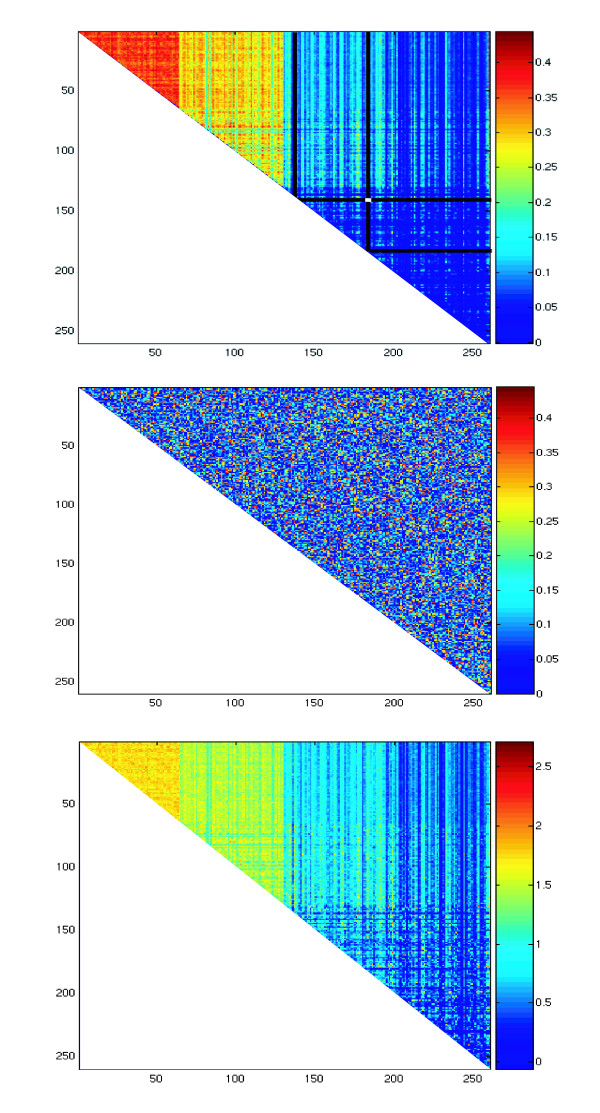
**Plots of MI values for an alignment**. The axis values represent the sites in the alignment, and the values in the matrix, depicted in color, are the MI values. a) top of page: Standard MI plot of a given alignment. The superimposed black lines are the matrix cells used for RCW the cell in white (under the black lines) b) in the middle: Randomization of the plot in a). c) bottom of page: RCW MI of the same alignment.

When computing measures of similarity between sites, the pairs made of sites that have the same pattern will, of course, have high scores (let us not forget that coevolution will also yield the same pattern of conservation). The chance of pairs of non-coevolving sites having a high score will then increase with the chance of two sites sharing the same pattern of conservation. Therefore, sites that have a common pattern of conservation have much higher chance of causing false positives pairs. This can be seen when looking at a MI plot between all sites. On figure 2a, we have a MI plot of an alignment of 256 taxa, without coevolving pairs, with 4 black lines superimposed on it (lets ignore these lines for the moment). On figure 2b, we have a random shuffling of the cells from the MI plot. Two things can be observed in figure 2a. First, that quickly evolving sites have, on average, higher MI values (sites on the left are the quickest, on the right the slowest evolving ones). That is evident in the orange triangle on the top-left corner of the matrix, followed by the yellow segment and finally the two blue ones. Secondly, figure 2a has much more row-and-column dependency. That translates to some lines and rows having many high scoring cells, while others have mostly low scoring cells. That can be perceived by looking at figure 1a and really "seeing" rows and columns. That is not possible to do in figure 2b. The row-and-column effect is caused by sites with a common pattern of conservation scoring high against each other – these sites are the ones that evolve more "accordingly" to the phylogenetic tree. It thus seem logical to weight each site pair by the average score of the constituting sites (which are the cells under the black lines as an example in figure 2a), as in:

RCW(A;B)=MIijMI.j+MIi.+MIj.+M.i−2MIij2n−2
 MathType@MTEF@5@5@+=feaafiart1ev1aaatCvAUfKttLearuWrP9MDH5MBPbIqV92AaeXatLxBI9gBaebbnrfifHhDYfgasaacH8akY=wiFfYdH8Gipec8Eeeu0xXdbba9frFj0=OqFfea0dXdd9vqai=hGuQ8kuc9pgc9s8qqaq=dirpe0xb9q8qiLsFr0=vr0=vr0dc8meaabaqaciaacaGaaeqabaqabeGadaaakeaacqWGsbGucqWGdbWqcqWGxbWvcqGGOaakcqWGbbqqcqGG7aWocqWGcbGqcqGGPaqkcqGH9aqpdaWcaaqaaiabd2eanjabdMeajnaaBaaaleaacqWGPbqAcqWGQbGAaeqaaaGcbaWaaSaaaeaacqWGnbqtcqWGjbqsdaWgaaWcbaGaeiOla4IaemOAaOgabeaakiabgUcaRiabd2eanjabdMeajnaaBaaaleaacqWGPbqAcqGGUaGlaeqaaOGaey4kaSIaemyta0KaemysaK0aaSbaaSqaaiabdQgaQjabc6caUaqabaGccqGHRaWkcqWGnbqtdaWgaaWcbaGaeiOla4IaemyAaKgabeaakiabgkHiTiabikdaYiabd2eanjabdMeajnaaBaaaleaacqWGPbqAcqWGQbGAaeqaaaGcbaGaeGOmaiJaemOBa4MaeyOeI0IaeGOmaidaaaaaaaa@5AD5@

where MI.j denotes the sum of the Mutual Information matrix over all lines in column j. We call this measure RCW (Row-Column-Weighting). Performing RCW on figure 2a, yields figure 2c. It can be seen that the row-column dependency is now weaker, albeit still present, and that the highest values of RCW MI are not predominantly present in the quickest evolving sites, as they were in the figure 2a. The use of other functions besides the arithmetic average might improve the removal of the row-column dependency, but most of the remainder is due to the fact that it is a simultaneous bi-dimensional optimization. The weighting can also be performed excluding the top hits of every row/column, to accommodate for more than two-way coevolution.

As RCW is a weighting of an existing matrix, it is compatible with all matrix-based methods. We therefore tested RCW with all the remaining statistics presented in this work.

#### 5) Multi-dimensional amino acid representation (MDAR)

In previous work addressing the question of how to detect coevolution, there have been two main approaches to looking at the sequence data, namely one where the focus is on individual amino acids, and another where the focus is instead on groups of amino acids. In our view, both approaches have drawbacks. The first approach treats all the 20 × 19 possible amino acid substitutions as different. A huge amount of evidence in phylogenetic science points to this not being the case. For instance, serine is physico-chemically similar to threonine, and substitution with the former is in some sense similar to substitution with the latter. The second approach has two problems: it relies on just one out of the many possible ways of grouping amino acids, and it treats amino acids within any group as completely identical while amino acids in different groups are seen as entirely different.

We have tried to accommodate for the similarities between amino acids by representing each amino acid by a vector of length 20, each dimension being a measure of the similarity between that amino acid and each other amino acid. This vector is, in fact, a row from the BLOSUM62 matrix, which represents similarities between amino acids based on empirically observed substitution patterns. Therefore, each site in the alignment becomes a matrix instead of a vector. By representing amino acids in this multidimensional fashion we solve the above-mentioned problem on how to categorize amino acids, but we are left with the problem of measuring coevolution. Mutual Information and similar statistics have no equivalent in this framework. So to compare two sites we start by calculating the correlation between them. We do that by calculating the Mantel correlation between the two matrices that represent each site.

#### 6) Multi-dimensional amino acid representation vs tree

Finally, we also tried to incorporate the phylogenetic signal into the Multi-dimensional amino acid representation, as we suspected that sites with a strong phylogenetic signal would also be "correlated" to the phylogenetic tree, as suggested in Figure 1. Specifically, we used the distance matrix of the tree as a measure of phylogenetic signal, and normalized the Multi-dimensional amino acid representation by the Mantel correlation of each of its sites to the distance matrix, as in the formula below:

MDARvsTree=corr(a,b)corr(a,tree)⋅corr(b,tree)
 MathType@MTEF@5@5@+=feaafiart1ev1aaatCvAUfKttLearuWrP9MDH5MBPbIqV92AaeXatLxBI9gBaebbnrfifHhDYfgasaacH8akY=wiFfYdH8Gipec8Eeeu0xXdbba9frFj0=OqFfea0dXdd9vqai=hGuQ8kuc9pgc9s8qqaq=dirpe0xb9q8qiLsFr0=vr0=vr0dc8meaabaqaciaacaGaaeqabaqabeGadaaakeaacqWGnbqtcqWGebarcqWGbbqqcqWGsbGucqWG2bGDcqWGZbWCcqWGubavcqWGYbGCcqWGLbqzcqWGLbqzcqGH9aqpdaWcaaqaaiabdogaJjabd+gaVjabdkhaYjabdkhaYjabcIcaOiabdggaHjabcYcaSiabdkgaIjabcMcaPaqaaiabdogaJjabd+gaVjabdkhaYjabdkhaYjabcIcaOiabdggaHjabcYcaSiabdsha0jabdkhaYjabdwgaLjabdwgaLjabcMcaPiabgwSixlabdogaJjabd+gaVjabdkhaYjabdkhaYjabcIcaOiabdkgaIjabcYcaSiabdsha0jabdkhaYjabdwgaLjabdwgaLjabcMcaPaaaaaa@653E@

where "corr" stands for Mantel Correlation. The real calculation was a transformation of the above one with the denominator scaled between 1 and 0 and then multiplied by the numerator, to prevent divisions by 0 or extremely low values.

### Evaluation of performance

To evaluate the performance of the methods on each alignment, we used the Area Under the ROC Curve (AUC) using[29]. The AUC is calculated from a ranked list, being threshold independent. It has the value 1 when all the positives are ranked higher than the negatives. In our case, the list is the output of each method, and with alignments of length 300 the list can extend up to 300 × 299/2 = 44850 pairs of sites. For simplicity, we considered only the 200 highest ranked pairs. In case some of the coevolving pairs were not present in the best 200, they were placed at the bottom of the list. This procedure means that the expected value of a random classifier is ≈ 0 (in most instances where AUC is applied, the whole list is used, the expected value of a random classifier is 0.5).

## Results and discussion

### General performance

As mentioned in the previous section, we simulated alignments containing 20 pairs of coevolving residues. This was done for 4 different numbers of taxa, for 3 different levels of coevolution and with data being simulated by 3 different methods. For each combination of these conditions, we generated 10 alignments (replicates) and analyzed each one of them by the different methods presented in Materials and Methods.

**Figure 3:**
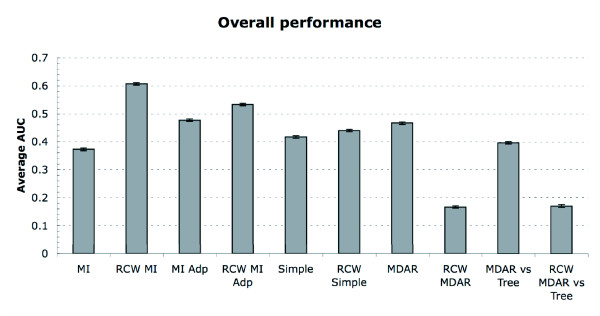
**Performance of each statistic**. This figure shows the average performance per each statistic (across all other factor conditions, measured in AUC). All differences are statistically significant at 0.01 with bonferroni correction, except the ones between MDAR and MI Adp, between RCW MDAR and RCW MDAR vs Tree and between MI and Simple

To compare the methods across all the different conditions, we performed a 4-way-Analysis of Variance (ANOVA) choosing as the dependent variable the Area Under the ROC Curve (AUC) of each alignment. The Analysis of Variance allows the comparison of the effects of all other variables (number of taxa, method of analysis, model of simulation, coevolution factor) on the dependent variable (the AUC), as well as checking if any of these variables interact with each other (for example, a given method of analysis being much better under a specific model of simulation). Given the large sample size, it is not surprising to find that all components had a significant effect (at 0.01) in the dependent variable. Moreover, interactions were also present, including the ones between the method and all other components. This means that the relative performance of the different methods is dependent on the simulation conditions. Indeed, the simulation covered a wide range of conditions; on one extreme (low coevolution factor, few taxa), it was very hard for even the best methods to find the coevolving residues. On the other extreme (high coevolution factor, many taxa), all coevolving residues consistently ranked top of the list. When comparing the performance of different methods, most differences were found to be significant (see figure 3 for details)

One can see that all non-Row-and-Column-Weighting methods outperform plain Mutual Information. One can also see that Row and Column Weighting improves all those methods which do not incorporate a multidimensional amino acid representation, in which case it totally ruins their performance. The most striking improvement is when Row and Column Weighting is done on a Mutual Information matrix, in which case the performance is 63% better than Mutual Information is on its own.

### Performance under different evolution models

**Figure 4:**
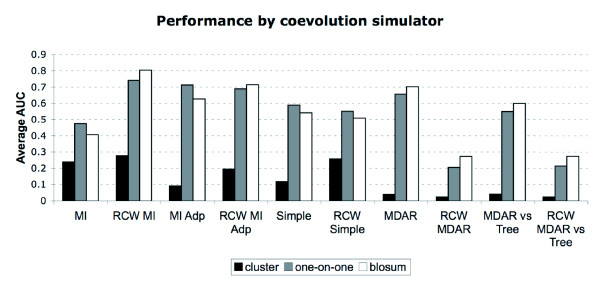
**Performance by coevolution simulator**. A histogram of average AUC using each of the 10 statistics. The 3 bars represent the 3 methods of coevolution used in the simulation.

We then compared the performances for each kind of simulation model (figure 4). In this way, we can see how the analysis methods perform under differently simulated data and, conversely, how important the choice of simulation model is when assessing performance of such methods. While datasets simulated by the models "one-on-one" and "BLOSUM" were relatively easy, the ones by "cluster" were much harder. Moreover, some statistics seem to perform relatively much better with data derived from some methods than from others. Row-and-Column-Weighting seems to be more effective under the "cluster" model, while the statistics incorporating multidimensional amino acid representation fare much worse. Row-and-Column-Weighed Mutual Information is the best method for data simulated under any of the three models.

### Combining different methods

**Figure 5:**
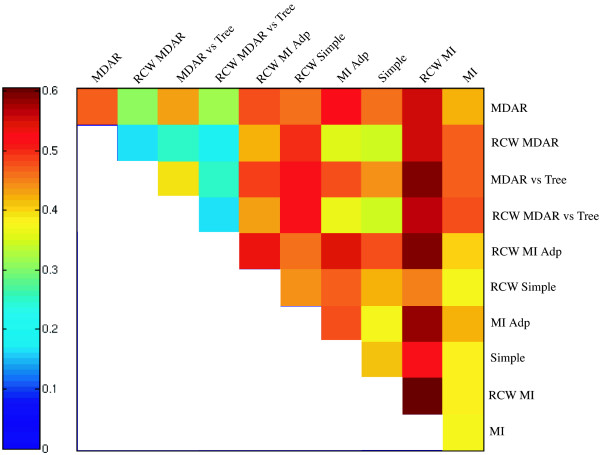
**Performance of all statistics and their interactions**. A matrix plot of the average AUC for all statistics and their interactions. Each axis has the 10 statistics, and the cells in the matrix represent the intersection of the two statistics represented in each axis.

We then proceeded to evaluate the intersections of the output lists given by each statistic, by means of their average AUC. From 10 original methods, we got 45 intersections. As one can see in figure 5, the intersection of output lists worked as expected: for all statistics except RCW MI, there was at least one intersection which outperformed it, even if not including the intersection with RCW MI. This seems to indicate that filtering out the negatives (specificity) is more important than picking out the positives (sensitivity), for improvement of performance. It can also be seen that methods with sub-optimal performance (*e.g*., MDRA vs. Tree) can give a positive contribution when combined with a sufficiently different statistic.

**Figure 6:**
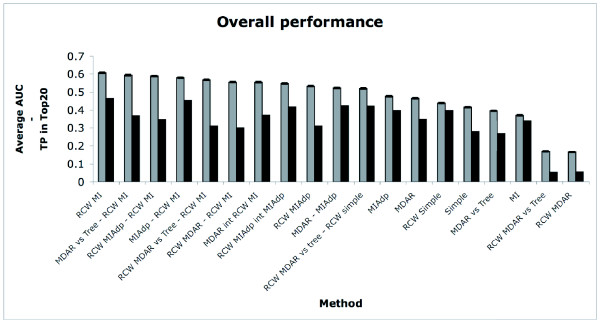
**Overall performance**. Histogram of the 11 best statistics plus the best original ones – ordered by decreasing AUC. Bars in grey are the average AUC, while the black bars are the average fraction of positives in the Top 20 positions of the output rank list. When comparing pairwise differences of AUCs, the best performing method is only statistically significantly better than the fifth best, and this one from the eight best.

We then ranked the 55 obtained statistics (10 original + 45 intersections). The outcome of the original statistics and the highest scoring intersections is shown in figure 6. We can see that RCW MI is the best statistic overall, closely followed by 6 statistics resulting from intersections between RCW MI and other methods. After them, come intersections including RCW MI Adp, MI Adp and MDRA, and RCW MI Adp alone. In 11th place comes the surprising intersection between RCW simple and RCW MDRA vs. Tree, showing how the intersection between two poor methods can yield good results.

Error bars, for an α = 0.05 and with Bonferroni correction, have also been added to the plot. These provide an indication of the level of randomness affecting our results, which we consider quite low. In addition to AUC, which is generally considered to be the best measure of overall performance, we have also used an alternative measure to analyze our results. Specifically, we counted the fraction of positives present in the best-ranked 20 pairs of sites (recall that there are 20 positives in each dataset). The average values are plotted as black bars, also on figure 6. We can see that this measure does not follow AUC closely, but the correlation is good enough for our purpose (RCW MI is the best statistic under both measures, and from the above 11 statistics, 5 will stay in the best 11 group). The major difference is that, under the fraction of positives in the best 20 pairs of sites, the best methods are not the intersections containing RCW MI.

### The effect of conservation on performance

**Figure 7:**
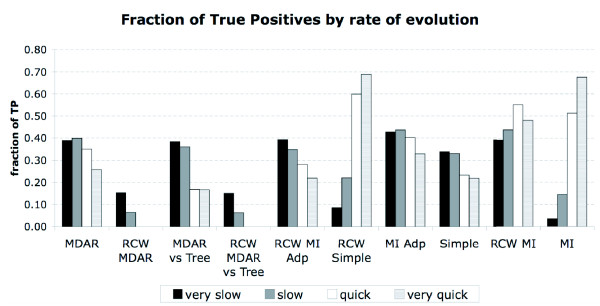
**Fraction of True Positives by rate of evolution**. Histogram of the fraction of true positives (for a threshold of the Top 20 hits) by statistic and rate of evolution. For each class of rate of evolution the maximum possible number of true positives (i.e., fraction of TP = 1) was 1800.

As shown in the work of [21], most methods are very dependent on conservation. To assess the effect conservation had on our results, we looked at true and false positives, taking into account their rates of evolution. It is immediately obvious that Mutual Information is biased towards quickly evolving sites (see Figure 7). One can also see that MI Adp, the "Simple" measure and Row and Column Weighting addresses that problem. Bias is hardly ever a desirable feature in a method, but we think that as truly coevolving sites are rarely the quickest evolving sites in an alignment, bias towards slowly evolving sites might prove much less harmful than the one showed by Mutual Information.

## Conclusion

Driven by the lack of a credible biological benchmarking system in which to test coevolution detection methods, we established a realistic and broad platform for benchmarking 2-way-coevolution at the protein sequence level. In our system, one can simulate coevolving sequences varying in the model of coevolution used, the strength of that coevolution, the evolutionary rate and the number of taxa. The output is then evaluated by comparing the Area Under the ROC Curve (AUC) or by the ratio between true positives and total positives.

We have shown how small differences in the benchmarking system can lead to disparate results and how strict and consensual one has to be in defining coevolution and the methods of its evaluation.

We have studied the effect of different rates of evolution between positions in one alignment, and verified that it is a major player in uncovering coevolution, and shown that, in this context, Mutual Information suffers from an "attraction" to quickly evolving sites that prevents it from becoming an effective coevolution detection measure.

To counter this effect, we have proposed several measures, including Row and Column Weighting of output matrices, which proved to be a strong improvement, increasing the accuracy of Mutual Information (measured by the Area Under the ROC Curve and in the conditions tested) by 63%. We have also included the concept of Multi-dimensional Amino acid Representation, which we believe has potential to be improved.

Finally, we have also compared the intersection of several measures, showing that this, in general, leads to an improvement in accuracy.

## Competing interests

The author(s) declare that they have no competing interests.

## Authors' contributions

RGO and AGP developed the concepts and ideas described in this work. RGO implemented them. Both RGO and AGP wrote the manuscript and approved this final version.
